# Establishment of vaginal microbiota composition in early pregnancy and its association with subsequent preterm prelabor rupture of the fetal membranes

**DOI:** 10.1016/j.trsl.2018.12.005

**Published:** 2019-05

**Authors:** Richard G. Brown, Maya Al-Memar, Julian R. Marchesi, Yun S Lee, Ann Smith, Denise Chan, Holly Lewis, Lindsay Kindinger, Vasso Terzidou, Tom Bourne, Phillip R. Bennett, David A. MacIntyre

**Affiliations:** a Imperial College Parturition Research Group, Division of the Institute of Reproductive and Developmental Biology, Imperial College London, London, UK; b Queen Charlotte's Hospital, Imperial College Healthcare NHS Trust, London, UK; c March of Dimes European Preterm Birth Research Centre, Imperial College London, London, UK; d Centre for Digestive and Gut Health, Imperial College London, London, UK; e School of Biosciences, Cardiff University, Cardiff, UK; f School of Medicine, Cardiff University, Cardiff, UK; g Chelsea & Westminster Hospital, Imperial College Healthcare NHS Trust, London, UK

**Keywords:** BMI, Body Mass Index, DNA, Deoxyribonucleic acid, ENA, European Nucleotide Archive, GA, Gestational Age, HCA, hierarchical cluster analysis, LDA, Latent discriminatory analysis, MR, Membrane Rupture, NHS, National Health Service, NICE, National Institute for Health and Care Excellence, PCR, Polymerase chain reaction, PPROM, Preterm prelabor rupture of the fetal membranes, PTB, Preterm birth, RDP, Ribosomal Database Project, SRA, Sequence Read Archive, STAMP, Statistical Analysis of Metagenomic Profiles, SOP, Standard Operating Procedure

## Abstract

Vaginal bacterial community composition influences pregnancy outcome. Preterm prelabor rupture of the fetal membranes (PPROM), which precedes 30% of all spontaneous preterm births, is associated with high vaginal bacterial diversity prior to rupture. The point at which vaginal bacterial diversity is established before PPROM is unknown. In this study, we use metataxonomics to longitudinally characterize the vaginal bacterial composition from as early as 6 weeks of gestation in women at high (*n* = 38) and low (*n* = 22) risk of preterm birth who subsequently experience PPROM and in women delivering at term without complications (*n* = 36). Reduced *Lactobacillus* spp. abundance and high diversity was observed prior to PPROM in 20% and 26% of women at low and high risk of preterm births respectively, but in only 3% of women who delivered at term. PPROM was associated with instability of bacterial community structure during pregnancy and a shift toward higher diversity predominately occurring during the second trimester. This was characterized by increased relative abundance of potentially pathogenic species including *Prevotella, Peptoniphilus, Streptococcus*, and *Dialister.* This study identifies reduced *Lactobacillus* spp. abundance and increasing vaginal bacterial diversity as an early risk factor for PPROM and highlights the need for interventional studies designed to assess the impact of modifying vaginal bacterial composition for the prevention of preterm birth.

**At a Glance Commentary**

**Richard G. Brown, et al.**

**Background**

Preterm birth (PTB) is the primary cause of death in children under 5yrs. Around 30% of cases are preceded by preterm prelabor rupture of fetal membranes (PPROM). A high diversity, *Lactobacillus* spp. deplete vaginal microbiome is a risk factor for PPROM, however it is unknown when in pregnancy this is established. By longitudinally characterizing vaginal composition from 6 weeks gestation, we show that PPROM is associated with bacterial community instability and shifts toward higher diversity, predominately during the second trimester.

**Translational Significance**

These results enable improved PTB risk stratification and targeted intervention strategies, which are reliant upon accurate identification of etiology.

Alt-text: Unlabelled box

## Background

Preterm birth (PTB) is the greatest challenge facing obstetrics in the modern era. It is the world's leading cause of childhood mortality and is associated with 80% of all neonatal morbidity^1^ resulting in major financial and emotional cost to families and society. Preterm prelabor rupture of the fetal membranes (PPROM) describes rupture of the fetal membranes prior to 37 weeks of gestation, before the onset of labor. PPROM is estimated to complicates 3% of pregnancies and is the largest contributor to spontaneous PTB, preceding 30% of cases,^2^ with 80% delivering within 9days^3^ and the overwhelming majority before 37 weeks.

Despite much research effort, the causes of PPROM are incompletely understood and the incidence continues to rise on a global scale.^4^ One widely held hypothesis is that a proportion of PPROM cases are caused by colonization of the vagina by pathogenic bacteria that activate the local innate immune system^2^, ^5^ precipitating an inflammatory cascade^6^, ^7^, ^8^, ^9^ that leads to untimely remodeling and disruption of fetal membrane architecture and eventually, premature rupture.^10^, ^11^, ^12^ Consistent with this hypothesis, recent studies using culture independent techniques have shown that reduced *Lactobacillus* spp. abundance and increased bacterial diversity is associated with PPROM and PTB.^13^, ^14^, ^15^ In contrast, healthy pregnancy is characterized by stable, low richness and low diversity community structures dominated by *Lactobacillus* spp.^16^, ^17^, ^18^ These findings concur with earlier culture-based studies that reported absence of *Lactobacillus* spp. and polymicrobial colonization of the vagina as risk factors for PPROM^19^ and PTB.^20^, ^21^

In a recent study of 250 pregnant women, we showed that vaginal bacterial composition characterized by *Lactobacillus* spp. depletion and high diversity, was detectable prior to the rupture of fetal membranes in approximately a third of cases.^13^ *Lactobacillus* spp. depletion and high diversity was not observed in women who subsequently delivered at term without complications. However, the point during the pregnancy when vaginal bacterial composition shifts toward a high-diversity state in women who subsequently PPROM remains unknown. To address this gap in our knowledge, we prospectively sampled over 1500 women with and without risk factors for PTB to identify 60 women who subsequently experienced PPROM. Vaginal microbiota compositionwas examined in these women from 6 to 36 weeks of gestation and compared to samples from women who subsequently delivered at term, matched for maternal age, BMI, and ethnicity. Our data demonstrates that vaginal bacterial communities deplete in *Lactobacillus* species and high in diversity are a risk factor for subsequent PPROM and predominately emerge during the second trimester.

## METHODS

### Study design

We performed a prospective cohort study of women with and without risk factors for preterm birth between January 2013 and November 2016. The study was approved by the National Health Service, National Research Ethics Service Committees for London–Stanmore (REC 14/LO/0328), and London-Riverside (REC 14/LO0199) areas. All ethical guidelines for human research were followed and participants provided written informed consent. Women without pre-existing risk factors for PTB were recruited from the early pregnancy unit of Queen Charlotte's Hospital, London (*n* = 1003). Women with existing risk factors (history of preterm birth, midtrimester loss (MTL) or large loop excision of the transformation zone (LLETZ) were recruited from the preterm birth surveillance clinics at Queen Charlotte's, St Mary's, and Chelsea and Westminster hospitals, London (*n* = 535). Exclusion criteria for both cohorts included women under 18years of age, multiple pregnancy, and sexual intercourse within 72 hours of sampling and HIV or Hepatitis C positive status.

Women were recruited upon presentation to the early pregnancy unit, (typically between 6 and 10weeks gestation),or at their initial appointment in the preterm birth surveillance clinic (12–14 weeks gestation). All women had cervicovaginal fluid sampled from the posterior vaginal fornix using a BBL™ CultureSwab MaxV Liquid Amies swab (Becton, Dickinson and Company, Oxford, UK). Swabs were placed immediately on ice before being stored at −80°C within 5minutes of collection. Repeat samples were taken where possible within the gestational time windows of; 12–17^+6^, 18–23^+6^, 24–29^+6^, 30–36^+6^ weeks^+days^ of completed gestation, however, not all women could be sampled at each of the prespecified timepoints. Detailed clinical and outcome data were collected for all participants. PPROM was defined as rupture of the fetal membranes, diagnosed by pooling of amniotic fluid on speculum examination, prior to 37 weeks gestation more than 24hours prior to spontaneous preterm delivery or clinically indicated delivery or induction of labor. Where speculum examination was equivocal, evidence of oligohydramnios or anhydramnios on ultrasonography, patient history and the decision of the attending clinician to administer steroids, erythromycin and induce labor at 34 weeks was accepted as a diagnosis of PPROM. Uncomplicated term deliveries without antepartum bleeding, antibiotic use, fetal growth restriction, pre-eclampsia, gestational diabetes, or other significant medical comorbidities were selected to match PPROM cases for age, ethnicity, and BMI.

### DNA extraction and sequencing of 16S rRNA gene amplicons

DNA extraction from vaginal swabs and assessment of DNA integrity by PCR amplification was performed as previously described.^22^, ^23^ The V1–V2 hyper variable regions of 16*S* rRNA genes were amplified for sequencing using forward and reverse fusion primers with the forward primer consisting of an Illumina i5 adapter (5′-AATGATACGGCGACCACCGAGATCTACAC-3′), an 8-base pair (bp) bar code, a primer pad (forward, 5′-TATGGTAATT-3′), and the 28F primer (5′-GAGTTTGATCNTGGCTCAG-3′).^24^ The reverse fusion primer consisted of an Illumina i7 adapter (5′-CAAGCAGAAGACGGCATACGAGAT-3′), an 8-bp bar code, a primer pad (reverse, 5′-AGTCAGTCAG-3′), and the 388R primer (5′-TGCTGCCTCCCGTAGGAGT-3′). Sequencing was performed at RTL Genomics (Lubbock, TX) using an Illumina MiSeq platform (Illumina Inc.) and the resulting sequence data analyzed using the MiSeq SOP Pipeline of the Mothur package.^25^ Sequence alignment was performed using the Silva bacterial database (www.arb-silva.de/), classification was performed using the Ribosomal Database Project database reference sequence files^26^ and the Ribosomal Database Project MultiClassifier script was used for determination of operational taxonomic unit taxonomies (phylum to genus). Species-level taxonomies were determined using USEARCH.^27^ To avoid sequencing bias, data were resampled and normalized to the lowest read count.

### Statistical analysis

Assessment of statistical differences between taxa was performed at genera and species level in the Statistical Analysis of Metagenomic Profiles software package .^28^ Samples were further classified into 3 groups according to Centroid linkage hierarchical clustering analysis of bacterial genera using a clustering density threshold of 0.75 with the 25most abundant genera displayed to facilitate visual presentation. Three separate clusters were identified and characterized on the basis of *Lactobacillus* spp. abundance into; dominant (75%–100% abundance), intermediate (50%–74% abundance), and deplete (0%–43% abundance). No samples were found to have relative *Lactobacillus* spp. abundance values between 43% and 49%.

Comparison of mean bacterial richness (species observed), diversity (inverse Simpson index) and relative abundances of bacterial genera was performed using nonparametric testing (Mann-Whitney *U*) and Fisher's exact test to compare frequency of vaginal microbiota compositions between the following groups; (1) women sampled at the last timepoint prior to PPROM vs gestational age matched samples from women delivering at term; (2) women sampled at 5 gestational age windows (6–11^+6^, 12–17^+6^, 18–23^+6^, 24–29^+6^, 30–36^+6^ weeks) who subsequently have PPROM vs those who subsequently deliver at term with no patient contributing more than 1 sample within any time window; (3) cases resulting in PPROM and delivery at less than 28, 34, or 37 weeks of gestation; (4) women with and without risk factors for PTB who experienced subsequent PPROM; (5) women with and without cervical cerclage who experienced PPROM; and (6) women who did or did not receive progesterone treatment prior to PPROM.

The relative risk of subsequent PPROM was calculated based using the relative abundance of lactobacilli (dominant, intermediate, depleted), the dominant *Lactobacillus* species and the presence of a nonlactobacilli dominated microbiome in women destined for term delivery or PPROM, at each of the 5 gestational age windows.

The LEfSe method^29^ was used to identify differentially abundant taxonomic features between the PPROM and term delivery groups. An *α* value of 0.05 was used for factorial Kruskal-Wallis test between classes, and a minimum threshold of 2.0 was used for logarithmic latent discriminatory analysis score for discriminative features. The significance of differences between percentage abundance of bacterial genera identified as discriminatory between the 2 outcome groups by LEfSe was calculated by Mann-Whitney.

To assess the dynamics of vaginal bacterial communities across pregnancy, longitudinal samples for pregnancies culminating in term delivery and PPROM were analyzed. The dominant microbiota and *Lactobacillus* spp. abundance for each sample prior to term delivery (62 samples, 16 term deliveries) and PPROM (167 samples, 45 PPROM cases) were highlighted and transitions between dominant microbiota and different abundances of *Lactobacillus* spp. examined.

## RESULTS

### Characterization of study population

**Fig 1:**
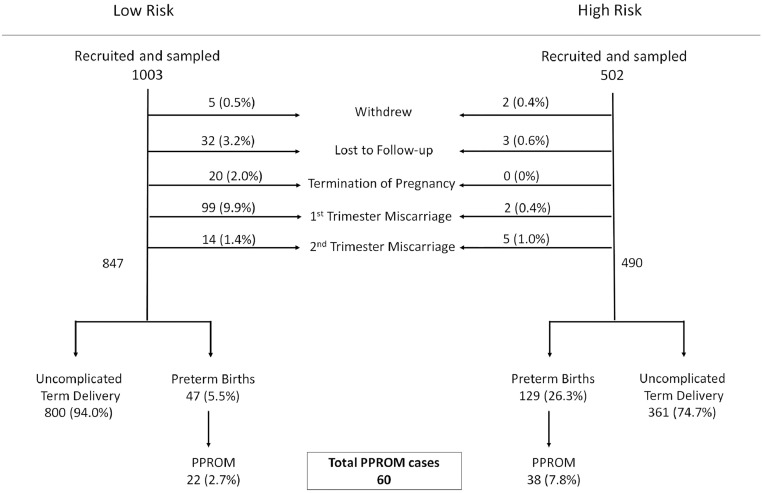
Prospective recruitment of low-risk and high-risk pregnant women. One thousand five hundred and fifty patients were recruited prospectively, yielding 60 cases of PPROM. PPROM, preterm prelabour rupture of the fetal membranes.

Between March 2013 and November 2016, 6324 women were seen Early Pregnancy Assessment Unit of Queen Charlotte's Hospital with 2667 meeting the inclusion criteria and 1048 being recruited, resulting in a participation rate of 38%. There were 5 withdrawals, 32 losses to follow-up, 20 terminations of pregnancy, 99 first trimester miscarriages and 14 second trimester miscarriages resulting in 847 ongoing singleton pregnancies. The large majority (800) delivered at term (94%), 47 delivered preterm (5.5%) of which 22 were spontaneous preterm deliveries (2.6%) and 23 experienced PPROM (2.7%). Of the 23 cases that experienced PPROM, 1 participant did not have samples collected. Simultaneously between January 2013 and November 2016, 502 women with risk factors for PTB including previous PTB, previous midtrimester spontaneous miscarriage, previous rescue or ultrasound indicated cervical cerclage and previous LLETZ procedure, were recruited from the preterm birth surveillance clinics at Queen Charlotte's, St Mary's, and Chelsea and Westminster hospitals. These women were recruited from 810 eligible participants resulting in a recruitment rate of 60%. Of these, 2 withdrew from the study, 3 were lost to follow-up and 7 experienced miscarriage resulting in 490 pregnancies that entered the third trimester. The majority (361) delivered at term, (75%) and of the 129 preterm births (26%), 38 (7.8%) cases experienced PPROM. Thus a total of 60 PPROM cases combined from the 2 cohorts were selected for sequencing (Fig 1) and were compared to 40 matched controls at a minimum ratio of 2:1 (cases:controls). Control women were matched for maternal age, gestational age at sampling, BMI, and ethnicity and included those without antepartum bleeding, antibiotic use, fetal growth restriction, pre-eclampsia, gestational diabetes or other significant medical comorbidities. Exclusion of 4 control samples due to amplification failure or low sequence reads resulted in a final sample size of 36 (TableI).

**Table I:** Clinical and demographic characteristics of the study cohort

	Uncomplicated term delivery	Pre-PPROM	*P* value
Total number	36	60	
Total number of samples	82	172	
Age (y)	33.3 (31.9–34.6)	33.8 (31.2–34.3)	0.70
BMI	24.8 (22.9–26.6)	24.5 (23.5–25.6)	0.82
Smoking status	1 (3%)	4 (7%)	0.65
Ethnicity			
Caucasian	18 (50 %)	31 (52%)	0.78
Black	9 (25%)	13 (21%)	
Asian	9(25%)	16 (27%)	
GA at PPROM (wk)		30^+5^ (29^+1^–32^+2^)	
GA at sample (wk)	24+6 (24^+2^–27^+3^)	25+5 (24–28^+2^)	0.78
GA at Delivery(wk)	39^+3^ (38^+4^–40^+4^)	32^+3^ (31^+1^–33^+5^)	<0.0001
Birth weight (g)	3342 (3094–3589)	2033 (1837–2230)	<0.0001

*Abbreviations:* BMI, body mass index; GA, gestational age; MR, membrane rupture; PPROM, preterm prelabour rupture of the fetal membranes.

Data presented as median (interquartile range) or number (%).

*P* values: *t* test/ Mann-Whitney *U* (depending upon distribution), Fisher's exact for proportional data.

A total of 254 swab samples were analyzed providing 4,593,060 high quality reads with an average read count of 18,083 per sample. After removal of singletons and rare operational taxonomic units (OTUs) (defined as <10 reads per sample) a total of 37 and 123 taxa were identified in control and subsequent PPROM samples respectively (Sup. Fig. 1). Hierarchical clustering of relative abundance data from the top bacterial genera (accounting for >95% of all sequence reads), permitting samples to be classified into 3 vaginal microbiota groups characterized by the relative abundance of *Lactobacillus* spp. and termed *Lactobacillus* spp. dominant (75%–100%, abundance), intermediate (50%–74%, abundance) and depleted (0%–43%, abundance) (Sup Fig 2).

### Vaginal microbiota composition exhibits variation by ethnicity

The final sample prior to PPROM (*n* = 60) and gestational age matched samples (*n* = 36) from women delivering at term were separated on the basis of ethnicity to examine vaginal bacterial composition in women of Caucasian, Asian, and Black ethnicities. Each of the predominant *Lactobacillus* spp. (*L. crispatus, L. iners, L. jensenii*, and *L. gasseri)* were identified in women of Asian and Caucasian ethnicity, but *L. gasseri* was not present in women of black ethnicity as previously reported in low risk pregnancies from the same background population.^17^ A *Lactobacillus* spp. deplete microbiome was not observed in Asian or Caucasian women delivering at term, but was present in 1/8 black women. Richness and diversity measures were comparable between ethnic groups in women delivering at term. In women with subsequent PPROM there was an increase in the number of women with a *Lactobacillus* spp. depleted vaginal microbiome across all ethnicities, with the greatest increase of *Lactobacillus* spp. deplete communities in black women (Sup Fig 3, Sup Table 1).

### Reduced *Lactobacillus* spp. abundance and increased richness is associated with subsequent PPROM

**Fig 2:**
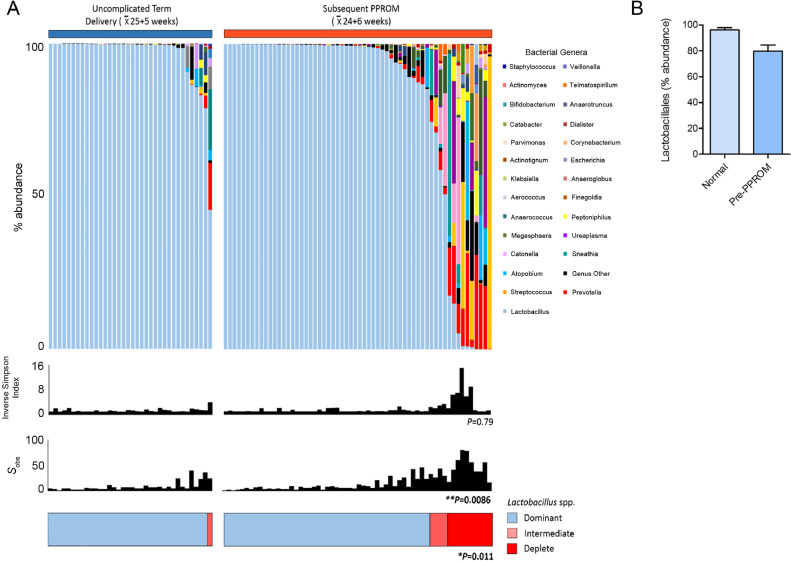
Women destined to experience PPROM exhibit reduced *Lactobacillus* spp. abundance and increased richness. **(A)**. Stacked bar chart displaying percentage abundance of the top 25 bacterial genera, *Lactobacillus* spp., diversity and richness of vaginal bacterial communities comparing the last sample taken before PPROM (*n* = 60) and samples taken at a similar gestation from women subsequently delivering at term matched for age, BMI and ethnicity (*n* = 36). Samples prior to PPROM exhibit significantly higher richness (*P* = 0.0086, Mann-Whitney) and a significantly higher proportion of women with *Lactobacillus* spp. intermediate or deplete communities (<75% abundance) *P* = 0.011, Fisher's Exact. **(B)**. Total abundance of *Lactobacillus* spp. is significantly higher on average in pregnancies delivering at term *P* = 0.016, Mann-Whitney *U* test. BMI, body mass index; PPROM, preterm prelabour rupture of the fetal membranes.

The last sample obtained before membrane rupture from the 60 PPROM cases was compared to samples taken from the term delivery group (*n* = 36) matched for gestational age at sampling, maternal age, BMI, and ethnicity (TableI). The average gestation of the last sample before PPROM was 24^+6^ weeks compared to 25^+5^ weeks for those delivering at term (*P* = 0.78, Mann-Whitney). The great majority of women (35/36, 97%) who delivered at term had a vaginal microbiome with >75% abundance of *Lactobacillus* spp. and 83% (30/36) had *Lactobacillus* spp. abundance above 98%. Samples obtained prior to PPROM were comparatively enriched for intermediate or *Lactobacillus* spp. depleted communities (PPROM; 14/60, 23% vs Control; 1/36, 3%, *P* = 0.011, Mann-Whitney), decreased total *Lactobacillus* spp. abundance (PPROM; 79% vs Control; 96%, *P* = 0.016 Mann-Whitney) and increased richness (total number of species observed PPROM; 65vs Control; 10, *P* = 0.0086) (Fig 2).

**Fig 3:**
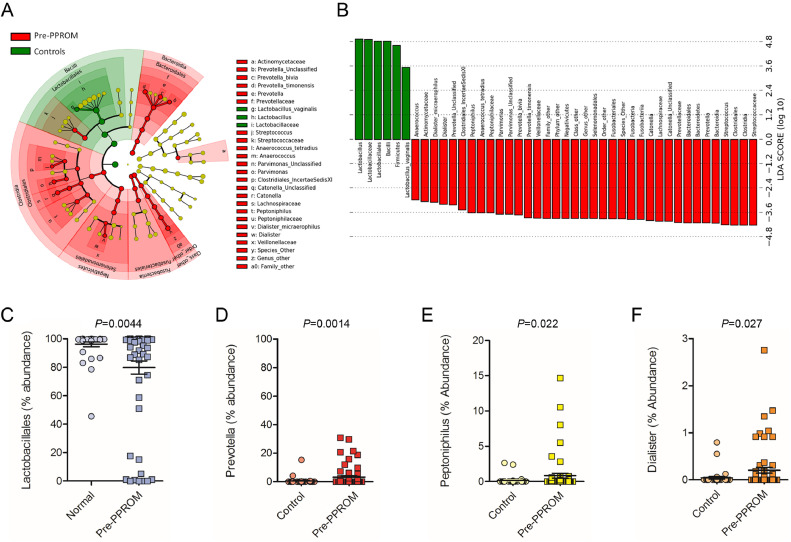
Bacterial taxonomic groups discriminate between normal term delivery and women destined to experience PPROM. (**A**) Cladogram describing differentially abundant vaginal bacterial clades and nodes observed between women subsequently experiencing normal term delivery or PPROM as identified using LEfSe analysis. (**B**) The effect size for each differentially abundant species was estimated using LDA. Vaginal microbiota of patients prior to PPROM was enriched with *Bacteroidales, Fusobacteriales*, and *Clostridiales* whereas those with a term delivery were comparatively enriched in *Lactobacillales*. Comparison of relative abundance across the 4 differentially expressed bacterial genera showing **(C)** reduced *Lactobacillales* (*P* = 0.0044) and **(D)** increased *Prevotella* (*P* = 0.0014), **(E)***Peptoniphilus* (*P* = 0.022) and **(F)***Dialister* (*P* = 0.0227, Mann-Whitney *U*, 2-tailed) in women prior to membrane rupture compared to term delivery control_._ LDA, latent discriminatory analysis; PPROM, preterm prelabour rupture of the fetal membranes.

LEfSe analysis of differentially abundant taxonomic features between PPROM and term delivery groups identified bacteria from the genera *Prevotella, Peptoniphilus, Dialister, Streptococcus, Catonella, Parvimonas*, and *Anaerococcus* as being discriminatory for pre-PPROM while *Lactobacillus* spp. were positively associated with term delivery (Fig 3). Percentage abundance of bacteria belonging to the genera *Prevotella* (*P =* 0.0014*), Peptoniphilus* (*P =* 0.022) and *Dialister* (*P =* 0.027) were significantly higher in samples pre-PPROM whereas *Lactobacillus* spp. were significantly reduced compared to term controls (*P* = 0.016; Fig 3). These corresponded at species level to *Prevotella bivia, Prevotella timonensis,* and *Dialister micraerophilus. Lactobacillus vaginalis* was associated with term delivery, but was only present in low abundance, comprising a maximum of 8.5% of sequence reads, and was always found in combination with other *Lactobacillus* species.

### Assessment of vaginal microbiota composition associated with PPROM risk across gestation

**Fig 4:**
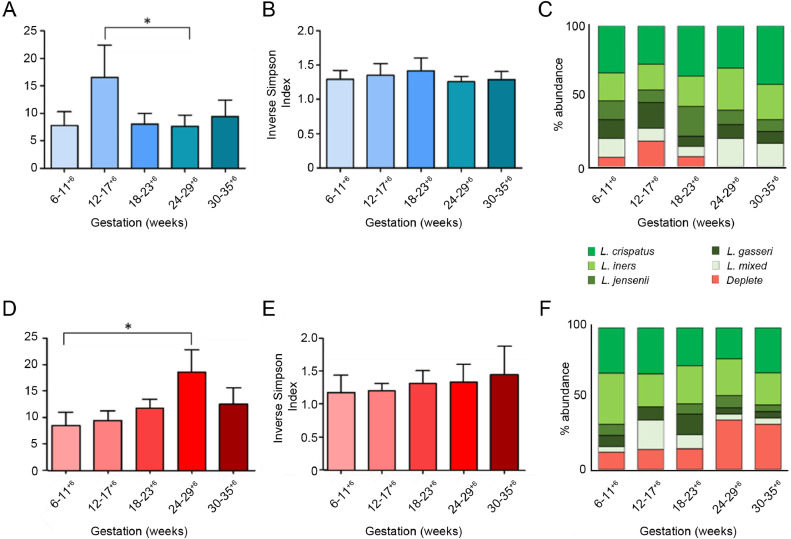
Bacterial richness and the proportion of women with a *Lactobacillus* spp. deplete microbiome increases with gestation in pregnancies destined for PPROM. Richness (species observed), diversity (Inverse Simpson Index) and *Lactobacillus* spp. abundance of vaginal bacterial communities sampled at 6–11^+6^, 12–17^+6^, 18–23^+6^, 24–29^+6^, 30–36^+6^ weeks gestation in women with subsequent term delivery and PPROM. **(A)** Richness was significantly higher at 12–17^+6^ weeks compared to 24–29^+6^ weeks in women with uncomplicated term deliveries (*P* = 0.03, Mann-Whitney). **(D)** Conversely in women with subsequent PPROM richness was significantly higher at 24–29^+6^ in comparison to 6–17^+6^ weeks (*P* = 0.02, Mann-Whitney) **(B, E)** Diversity remained stable across gestation. There were no statistically significant differences in *Lactobacillus* spp. abundance across gestation. **(C)** In women with subsequent term delivery all samples were *Lactobacillus* spp. dominant above 24 weeks. **(D)** In women with subsequent PPROM the proportion of women with *Lactobacillus* spp. deplete communities increases above 24 weeks. PPROM, preterm prelabour rupture of the fetal membranes.

Longitudinal analyses showed that uncomplicated term delivery was associated with stable alpha diversity (Inverse Simpson index; *P* = 0.33, Kruskal Wallis, Dunn's post hoc), *L crispatus* dominance and reduced richness between 12–17^+6^ and 24–29^+6^ weeks (*P* = 0.03, Kruskal Wallis, Dunn's post hoc) compared to women who subsequently had PPROM (Fig 4). In contrast, subsequent PPROM was associated with a significant increase in richness between 6–11^+6^ and 24–29^+6^ weeks (*P* = 0.02, Kruskal Wallis, Dunn's post hoc) (Fig 4, Sup Table 2).

**Fig 5:**
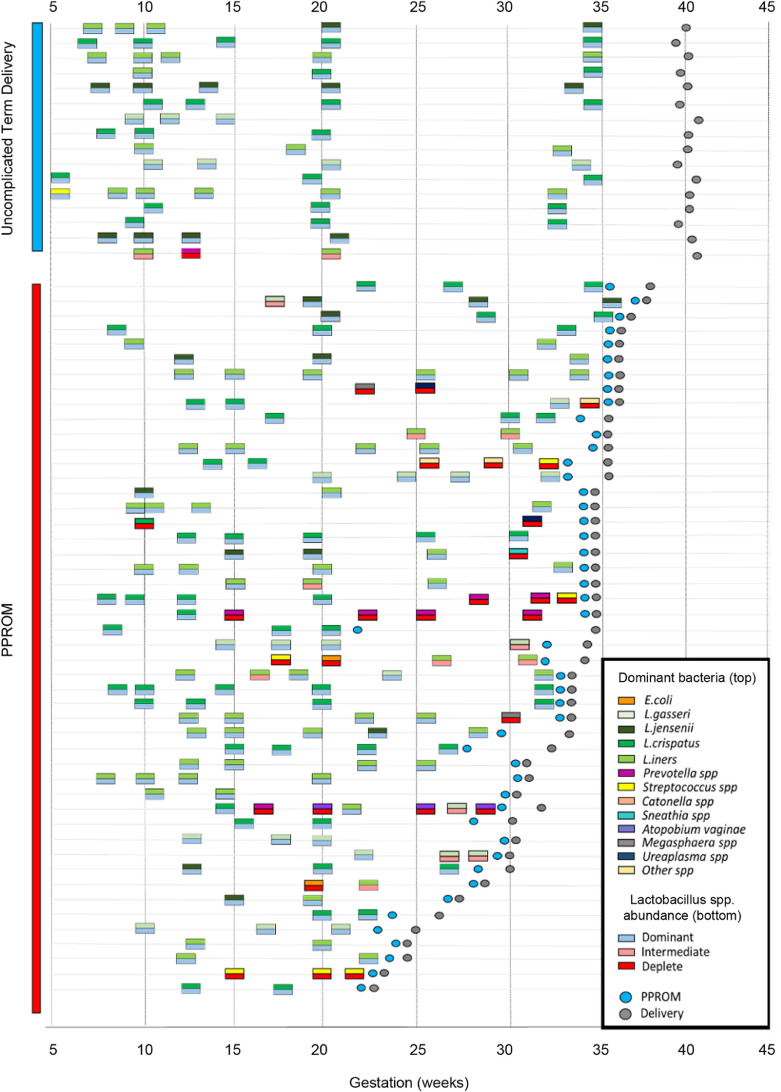
Vaginal bacterial communities demonstrate instability prior to PPROM. Longitudinal profiling of vaginal bacterial communities displaying the dominant bacterial species (top) and *Lactobacillus* abundance state (bottom) for each sample from women with subsequent term delivery and PPROM who were sampled on 2 or more occasions antenatally. Gestation at PPROM is denoted by the blue circle while delivery is denoted by the gray circle. The vaginal bacterial community remained stable throughout pregnancy for the majority (13/16, 81%) of women with subsequent term delivery. In contrast 37% of women with PPROM experienced transition from one state to another at some point prior to membrane rupture. PPROM, preterm prelabour rupture of the fetal membranes.

A total 45 cases of subsequent PPROM had multiple antenatal samples and were compared to 16 individuals with term delivery. The majority (13/16, 81%) of individuals delivering at term displayed a vaginal microbiome that was dominated by a single *Lactobacillus* spp. and remained stable throughout the pregnancy with no detected transition events recorded (Fig5). Only 2 women demonstrated transition from the microbiome present in the first trimester, transitioning from *L. iners* to *L. jensenii* and *L. crispatus* respectively prior to term delivery. There was a single case of term delivery where *Lactobacillus* spp. abundance was reduced and in which *L. iners* co-colonized with *Sneathia, Megasphaera,* and *Prevotella* spp. In contrast only 24/46 (56%) women with subsequent PPROM maintained a vaginal microbiome dominated by a single species of *Lactobacillus*, 7/46 (15%) transitioned between different *Lactobacillus* spp. maintaining an overall dominance of *Lactobacillus* spp. Within the PPROM group, 22% (10/46) transitioned between a *Lactobacillus* spp. dominant and deplete community structure dominated by genera including *Streptococcus, Prevotella, Atopobium,* and *Megasphaera.* Overall 17/46 (37%) of women demonstrated some form of transition from one dominant taxon to another prior to membrane rupture and there were a greater number of individuals with a microbiome persistently devoid of *Lactobacillus* spp. (4/46, 9%) within the PPROM group (Fig 5; update figure numbers).

### Relationship between vaginal microbiota composition and relative risk of PPROM

**Fig 6:**
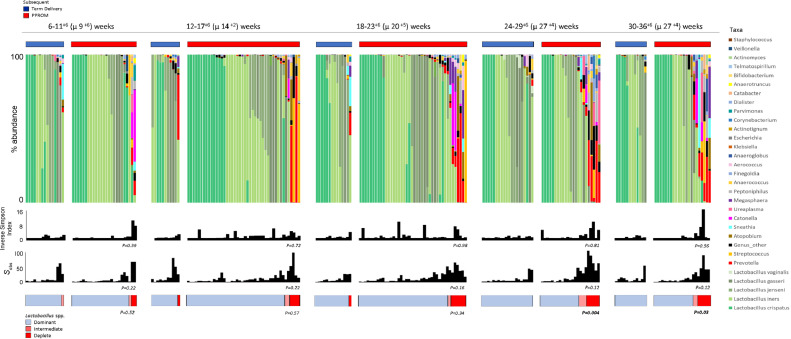
Vaginal bacterial communities with reduced *Lactobacillus* spp. abundance are associated with subsequent PPROM from 24 weeks of gestation. Stacked bar chart displaying percentage abundance of the top 25 bacterial genera, *Lactobacillus* spp., diversity and richness of vaginal bacterial communities at 6–11^+6^, 12–17^+6^, 18–23^+6^, 24–29^+6^, 30–36^+6^ weeks gestation for women with subsequent term delivery and PPROM. The proportion of women with a vaginal microbiome with reduced *Lactobacillus* spp. abundance is significantly higher in the subsequent preterm membrane rupture group compared to term delivery at 24–29^+6^ (*P* = 0.004) and 30–36^+6^ (*P* = 0.03, Fisher's Exact). PPROM, preterm prelabour rupture of the fetal membranes.

**Fig 7:**
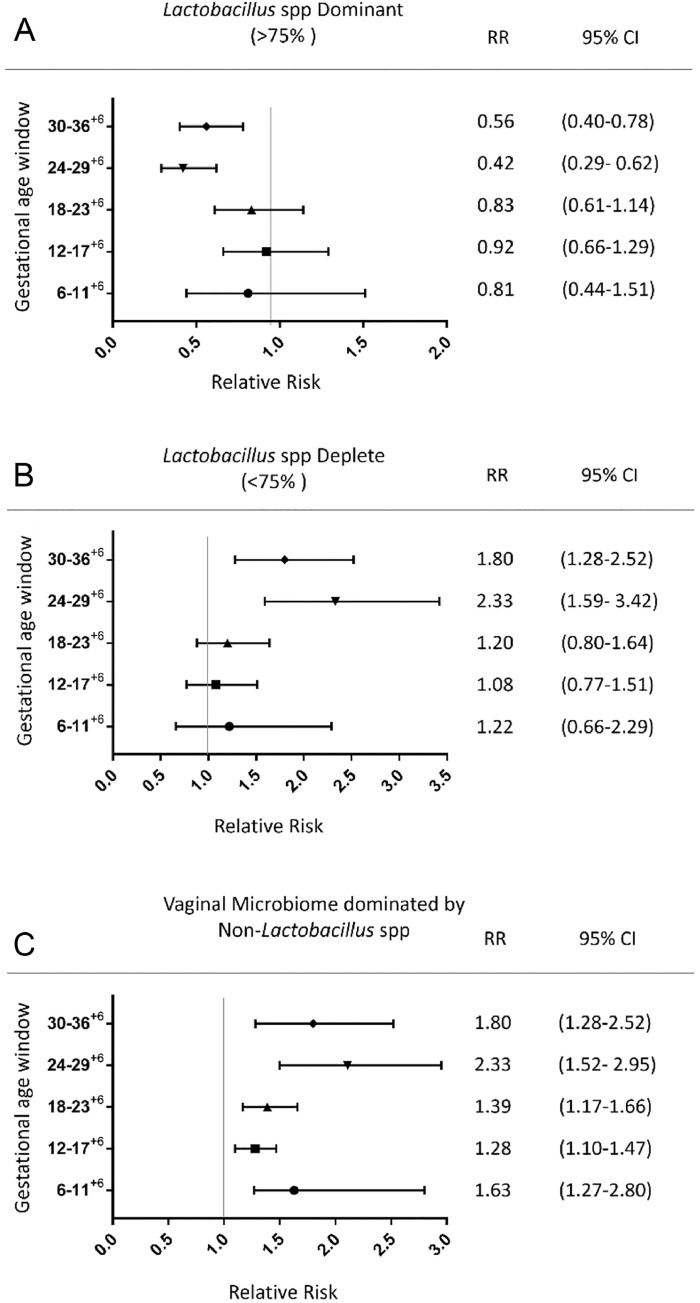
Vaginal bacterial communities devoid of *Lactobacillus* spp. from 6 weeks and reduced *Lactobacillus* spp. abundance beyond 24 weeks are risk factors for subsequent PPROM. Forest plot, relative risk of subsequent PPROM based upon the composition of the vaginal microbiome at each gestational age window. **(A)** Bacterial communities dominated by *Lactobacillus* spp. beyond 24 weeks reduce the relative risk of subsequent PPROM. **(B)** Bacterial communities with reduced *Lactobacillus* spp. abundance beyond 24 weeks increase the relative risk of PPROM. **(C)** A vaginal microbiome dominated by non-*Lactobacillus* spp. is associated with increased risk of PPROM at all gestational ages. PPROM, preterm prelabour rupture of the fetal membranes.

Calculation of relative risk of subsequent PPROM was performed for each time window based on the predominant *Lactobacillus spp*., overall abundance of *Lactobacillus* spp. (dominant, intermediate, deplete) and the presence of a vaginal microbiome dominated by bacterial genera other than Lactobacilli (Fig 6 and 7). A vaginal microbiome with reduced *Lactobacillus* spp. abundance (<75%) was associated with increased relative risk; 2.56 (1.66–3.88) and 2.34 (1.59–3.42) at 24–29^+6^ and 30–35^+6^ weeks respectively. While vaginal bacterial communities dominated by *Lactobacillus* spp. beyond 24 weeks were associated with a reduction in PPROM risk 0.39 (0.26–0.60), RR-0.43 (0.29–0.63) at 24–29^+6^ and 30–35^+6^ weeks respectively (Fig 7). Vaginal bacterial communities dominated by any species other than *Lactobacillus* was associated with subsequent PPROM at all gestational time windows (RR 1.63 (1.27–2.80), 1.28 (1.10–1.47), 1.39 (1.17–1.66), 2.11 (1.52–2.95), 1.8 (1.28–2.52); Fig 7).

### High-risk vaginal microbiota in low- and high-risk pregnancies

**Fig 8:**
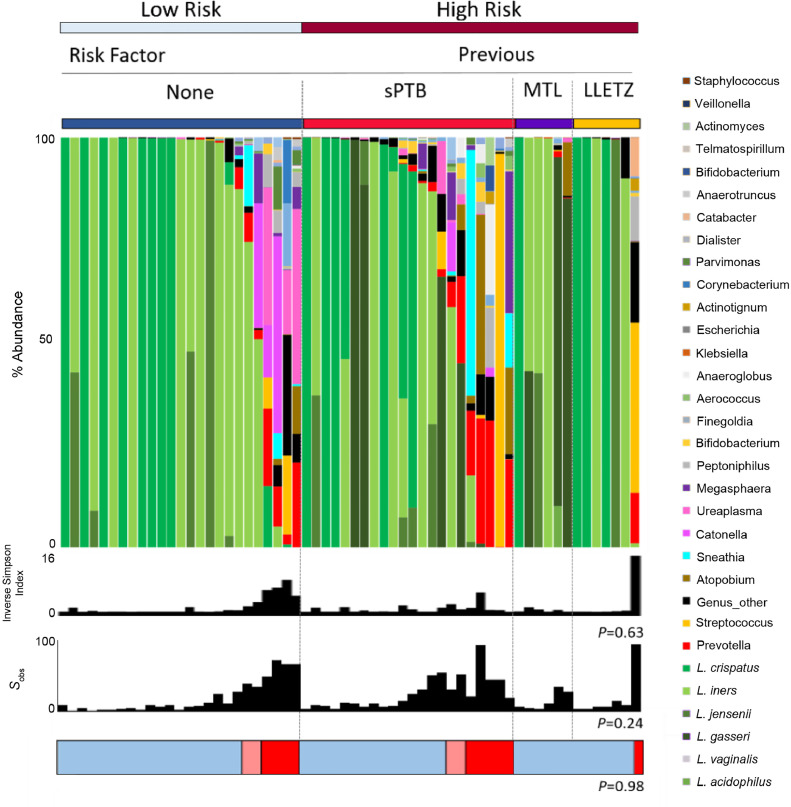
Vaginal bacterial communities with reduced *Lactobacillus* spp. abundance are equally distributed between women considered at low and high risk of PTB. Stacked bar chart displaying percentage abundance of the top 25 bacterial genera, *Lactobacillus* spp., diversity and richness of vaginal bacterial communities for the last sample taken before PPROM for women considered low and high risk of PTB in the index pregnancy. The proportion of women with bacterial communities deficient in lactobacilli is similar for women considered low and high risk for PTB (5/25, 20% vs 9/35, 26%, *P* = 0.76, Fisher's exact).

In women with no pre-existing risk factors for PTB and considered low risk in the index pregnancy, 20% (5/25) had *Lactobacillus* spp. depleted communities prior to PPROM compared to 26% (9/35) of those at high risk of PTB (*P* = 0.76, Fisher's exact). Women with a previous history of PTB were the most likely to have vaginal bacterial communities with *Lactobacillus* spp. abundance <75% (36%, 8/22). However, only 1/7 (14%) women with a previous LLETZ, and 0/6 (0%) of women with history of a midtrimester miscarriage had a microbiome deplete in *Lactobacillus* spp. during their pregnancy (Fig 8).

### Impact of intervention on high-risk vaginal microbiota associated with subsequent PPROM

Over a third (38%, 23/60) of women with subsequent PPROM had a cervical cerclage, the majority of which were vaginally inserted Mersilene cerclages (14/23, 61%) followed by vaginal loop Nylon (6/23, 26%,) and abdominal cerclages (3/26, 13%). Mersilene cerclage was associated with the greatest proportion of women with reduced *Lactobacillus* spp. abundance (25%, 5/14). Only one woman had reduced lactobacilli in the Nylon cerclage group and all of the abdominal cerclage cases were associated with vaginal lactobacilli dominance. The proportion of women with reduced *Lactobacillus* spp. abundance before PPROM who did not have a cervical cerclage was similar (22%, 8/37; Sup. Fig 5) and vaginal progesterone treatment was not associated with increased *Lactobacillus* spp. dominance (*P* = 0.74, Fisher's Exact; Sup. Fig 5).

## DISCUSSION

Our study examines the emergence during pregnancy of an association between vaginal bacterial communities depleted in *Lactobacillus* spp. and increased risk of PPROM in women at both high and low risk of spontaneous PTB. This work has important potential implications for future stratification of PTB risk and targeted, preventative interventions, the success of which are highly reliant upon accurate identification of the underlying etiology.

*Lactobacillus* spp*.* deplete communities in women destined for PPROM were maximal at 24–29^+6^ weeks gestation and were associated with enrichment of bacteria such as *Prevotella, Streptococcus, Peptoniphilus, Ureaplasma,* and *Dialister* spp. These bacteria are well described opportunistic colonizers capable of up regulating the expression of metalloproteinases^30^, ^31^ and proinflammatory cytokines^32^ while reducing the inhibitory effect of tissue inhibitor metalloproteinases (TIMPs).^33^ In contrast, women with subsequent term delivery experienced reduced richness as gestation progressed with all samples beyond 24 weeks dominated by *Lactobacillus* spp*.* These trends are consistent with a recent meta-analysis incorporating 4 independent studies profiling the vaginal microbiome during pregnancy, which reported that diversity increased throughout early pregnancy peaking between 20 and 29 weeks in women with subsequent PTB, but fell progressively across gestation in women with term deliveries.^34^ This time period coincides with a recently described “immune clock” during pregnancy that involves enhanced immunologic function, including heightened TLR4 response in neutrophils and up regulation of T-cells, between 20 and 30 weeks of gestation.^35^ This feature may represent a primed immunologic state that is sensitive to pathogenic vaginal bacteria during this phase of pregnancy. Previous studies have identified a dominance of *L. iners* at 16 weeks as a risk factor for subsequent PTB.^23^ *L. iners* was not associated with subsequent PPROM in this study, which may reflect different etiologies for PTB and PPROM, differences in patient populations or that women with an *L. iners* dominant microbiome at 16 weeks are more susceptible to bacterial instability and may develop a *Lactobacillus* spp. depleted microbiome in later pregnancy,^36^ prior to PPROM. The proportion of women with a *Lactobacillus* spp. deplete microbiome was particularly pronounced in women of Black ethnicity with 50% demonstrating low lactobacillus abundance prior to PPROM. This may imply that bacterial composition is of particular importance in black women who epidemiologically are at higher risk of PPROM {Savitz, 1991 #42} but this required further investigation.

Overall the composition of the vaginal microbiome prior to membrane rupture and delivery in extreme, very and late preterm cases was comparable which was surprising given the increased incidence of chorioamnionitis at extreme preterm gestations.^37^ This similarity may be caused by a shift toward reduced *Lactobacillus* spp. abundance observed following membrane rupture. We have previously demonstrated transition toward lactobacilli depleted communities enriched with pathobionts 48hours after PPROM and erythromycin treatment.^13^ Latency between PPROM and delivery tends to be prolonged at early gestations^3^ providing ample time for remodeling of vaginal bacterial communities with the infiltration of pathogens associated with ascending infection and chorioamnionitis.^13^ Overall average latency following PPROM was lower and the rate of chorioamnionitis higher in those women with a *Lactobacillus* spp. deplete microbiome prior to PPROM. This might reflect a vaginal microbiome with increased bacterial richness prior to PPROM leading to earlier delivery as a result of inflammatory activation and stimulation of preterm labor pathways or as a result of pre-existing vaginal pathobionts causing ascending infection and chorioamnionitis once the fetal membrane barrier is lost.

Differences in bacterial community stability in patients from low-and high-risk backgrounds for PTL were also observed with women who experience subsequent PPROM exhibiting increased transition from one dominant species or taxa to another prior to membrane rupture, consistent with recent reports of an association between decreased vaginal bacterial community stability and increased risk of PTB in high risk black populations.^38^

Women with a history of preterm birth were more likely to display a vaginal microbiome with reduced *Lactobacillus* spp. abundance when compared to women with a previous LLETZ or MTL. This difference indicates the existence of a PPROM etiology independent of host-bacteria interactions and instead is caused by mechanical cervical insufficiency in women with a history of LLETZ or MTL.^39^ Instead of antibiotics, such women may respond well to restoring cervical integrity with the appropriate cervical cerclage, whereas more nuanced approaches incorporating modification of the vaginal microbiota may be more effective for women with a previously unexplained PTB.

Consistent with treatment practices, a proportion of women who subsequently experienced PPROM received vaginal progesterone or cervical cerclage in an attempt to prevent PTB within the study population. A comparison between women who experienced PPROM with and without intervention showed a similar proportion of women with reduced *Lactobacillus* spp. abundance. The relationship between vaginal progesterone and the vaginal microbiome has been previously reported in women delivering at term and preterm.^15^ Mersilene cerclage was associated with a higher proportion of women with *Lactobacillus* spp. deficiency in comparison to nylon and abdominal cerclage. This finding is consistent with an earlier study that showed Mersilene cerclage induces vaginal dysbiosis accompanied by local inflammatory activation and early remodeling of the cervix.^15^ It is plausible that such a mechanism could lead to increased rates of PPROM in women receiving a Mersilene cerclage.

A limitation of our study is that examination of vaginal microbiota composition across patient groups preceding PPROM was performed using relative abundance comparisons determined by 16S rRNA gene sequencing. Future studies incorporating estimation of total bacterial load (eg, broad range 16S rRNA qPCR) could be performed to examine if this, in addition to compositional shifts, is a risk factor for subsequent PPROM.

In conclusion our study reveals that a vaginal microbiome depleted of *Lactobacillus* spp. is a risk factor for PPROM in roughly 25% of cases, independent of maternal characteristics and preterm birth risk. Cervicovaginal fluid can be easily sampled and with rapidly evolving technologies, quick and cost-effective point of care testing to assess *Lactobacillus* spp. abundance and the presence of pathobionts may be available in the near future.^40^ Unlike the contribution of genetic factors, antepartum hemorrhage and anatomical abnormalities, an unfavorable vaginal microbiome is a modifiable risk factor for PPROM. Identification of this subset of patients followed by manipulation of bacterial communities through a combination of antibiotic, prebiotic and probiotic therapies warrants further investigation and may represent a promising strategy for the reduction and/or prevention of PPROM and PTB.

## Appendix Supplementary materials

Image, application 1

## References

[1] WHO, World Health Organisation. Causes of child mortality. In: Global Health Observatory (GHO) Data. 2017. https://www.who.int/gho/child_health/mortality/causes/en/.

[2] Parry S., Strauss J.F. (1998). Premature rupture of the fetal membranes. N Engl J Med.

[3] Peaceman A.M., Lai Y., Rouse D.J., Spong C.Y., Mercer B.M., Varner M.W. (2015). Length of latency with preterm premature rupture of membranes before 32 weeks' gestation. Am J Perinatol.

[4] Liu L., Oza S., Hogan D., Perin J., Rudan I., Lawn J.E. (2015). Global, regional, and national causes of child mortality in 2000-13, with projections to inform post-2015 priorities: an updated systematic analysis. Lancet.

[5] Chandiramani M., Bennett P.R., Brown R., Lee Y., MacIntyre D.A. (2014). Vaginal microbiome-pregnant host interactions determine a significant proportion of preterm labour. Fetal Matern Med Rev.

[6] Kanayama N., Terao T., Horiuchi K. (1988). The role of human neutrophil elastase in the premature rupture of membranes. Asia-Oceania JObstet Gynaecol.

[7] Fortunato S.J., Menon R., Lombardi S.J. (2002). Role of tumor necrosis factor-alpha in the premature rupture of membranes and preterm labor pathways. Am J Obstet Gynecol.

[8] Shobokshi A., Shaarawy M. (2002). Maternal serum and amniotic fluid cytokines in patients with preterm premature rupture of membranes with and without intrauterine infection. Int J Gynaecol Obstet.

[9] Helmig B.R., Romero R., Espinoza J., Chaiworapongsa T., Bujold E., Gomez R. (2002). Neutrophil elastase and secretory leukocyte protease inhibitor in prelabor rupture of membranes, parturition and intra-amniotic infection. J Matern Fetal Neonatal Med.

[10] Fortner K.B., Grotegut C.A., Ransom C.E., Bentley R.C., Feng L., Lan L. (2014). Bacteria localization and chorion thinning among preterm premature rupture of membranes. PloS One.

[11] Goldenberg R.L., Culhane J.F., Iams J.D., Romero R. (2008). Epidemiology and causes of preterm birth. Lancet.

[12] Saigal S., Doyle L.W. (2008). An overview of mortality and sequelae of preterm birth from infancy to adulthood. Lancet.

[13] Brown R.G., Marchesi J.R., Lee Y.S., Smith A., Lehne B., Kindinger L.M. (2018). Vaginal dysbiosis increases risk of preterm fetal membrane rupture, neonatal sepsis and is exacerbated by erythromycin. BMC Med.

[14] DiGiulio D.B., Callahan B.J., McMurdie P.J., Costello E.K., Lyell D.J., Robaczewska A. (2015). Temporal and spatial variation of the human microbiota during pregnancy. Proc Natl Acad Sci USA.

[15] Kindinger L.M., MacIntyre D.A., Lee Y.S., Marchesi J.R., Smith A., McDonald J.A. (2016). Relationship between vaginal microbial dysbiosis, inflammation, and pregnancy outcomes in cervical cerclage. Sci Transl Med.

[16] Aagaard K., Riehle K., Ma J., Segata N., Mistretta T.A., Coarfa C. (2012). A metagenomic approach to characterization of the vaginal microbiome signature in pregnancy. PloS One.

[17] MacIntyre D.A., Chandiramani M., Lee Y.S., Kindinger L., Smith A., Angelopoulos N. (2015). The vaginal microbiome during pregnancy and the postpartum period in a European population. Sci Rep.

[18] Romero R., Hassan S.S., Gajer P., Tarca A.L., Fadrosh D.W., Nikita L. (2014). The composition and stability of the vaginal microbiota of normal pregnant women is different from that of non-pregnant women. Microbiome.

[19] McGregor J.A., French J.I., Seo K. (1993). Premature rupture of membranes and bacterial vaginosis. Am J Obstet Gynecol.

[20] Flynn C.A., Helwig A.L., Meurer L.N. (1999). Bacterial vaginosis in pregnancy and the risk of prematurity: a meta-analysis. J Fam Prac.

[21] Hillier S.L., Nugent R.P., Eschenbach D.A., Krohn M.A., Gibbs R.S., Martin D.H. (1995). Association between bacterial vaginosis and preterm delivery of a low-birth-weight infant. The Vaginal Infections and Prematurity Study Group. N Engl J Med.

[22] MacIntyre D.A., Chandiramani M., Lee Y.S., Kindinger L., Smith A., Angelopoulos N. (2015). The vaginal microbiome during pregnancy and the postpartum period in a European population. Sci Rep.

[23] Kindinger L.M., Bennett P.R., Lee Y.S., Marchesi J.R., Smith A., Cacciatore S. (2017). The interaction between vaginal microbiota, cervical length, and vaginal progesterone treatment for preterm birth risk. Microbiome.

[24] Sundquist A., Bigdeli S., Jalili R., Druzin M.L., Waller S., Pullen K.M. (2007). Bacterial flora-typing with targeted, chip-based Pyrosequencing. BMC Microbiol.

[25] Kozich J.J., Westcott S.L., Baxter N.T., Highlander S.K., Schloss P.D. (2013). Development of a dual-index sequencing strategy and curation pipeline for analyzing amplicon sequence data on the MiSeq Illumina sequencing platform. Appl Environ Microbiol.

[26] Wang Q., Garrity G.M., Tiedje J.M., Cole J.R. (2007). Naive Bayesian classifier for rapid assignment of rRNA sequences into the new bacterial taxonomy. Appl Environ Microbiol.

[27] Edgar R.C. (2010). Search and clustering orders of magnitude faster than BLAST. Bioinformatics.

[28] Parks D.H., Beiko R.G. (2010). Identifying biologically relevant differences between metagenomic communities. Bioinformatics.

[29] Segata N., Izard J., Waldron L., Gevers D., Miropolsky L., Garrett W.S. (2011). Metagenomic biomarker discovery and explanation. Genome Biol.

[30] Guan S.M., Shu L., Fu S.M., Liu B., Xu X.L., Wu J.Z. (2008). Prevotella intermedia induces matrix metalloproteinase-9 expression in human periodontal ligament cells. FEMS Microbiol Lett.

[31] Guan S.M., Shu L., Fu S.M., Liu B., Xu X.L., Wu J.Z. (2009). Prevotella intermedia upregulates MMP-1 and MMP-8 expression in human periodontal ligament cells. FEMS Microbiol Lett.

[32] Borgdorff H., Gautam R., Armstrong S.D., Xia D., Ndayisaba G.F., van Teijlingen N.H. (2016). Cervicovaginal microbiome dysbiosis is associated with proteome changes related to alterations of the cervicovaginal mucosal barrier. Mucosal Immunol.

[33] Nakata K., Yamasaki M., Iwata T., Suzuki K., Nakane A., Nakamura H. (2000). Anaerobic bacterial extracts influence production of matrix metalloproteinases and their inhibitors by human dental pulp cells. J Endod.

[34] Haque M.M., Merchant M., Kumar P.N., Dutta A., Mande S.S. (2017). First-trimester vaginal microbiome diversity: a potential indicator of preterm delivery risk. Sci Rep.

[35] Aghaeepour N., Ganio E.A., McIlwain D., Tsai A.S., Tingle M., Van Gassen S. (2017). An immune clock of human pregnancy. Sci Immunol.

[36] Verstraelen H., Verhelst R., Claeys G., Backer E., Temmerman M., Vaneechoutte M. (2009). Longitudinal analysis of the vaginal microflora in pregnancy suggests that L. crispatus promotes the stability of the normal vaginal microflora and that L. gasseri and/or L. iners are more conducive to the occurrence of abnormal vaginal microflora. BMC Microbiol.

[37] Rajan R, Menon V. Preterm premature rupture of membranes: correlates and pregnancy outcome in a tertiary care setting. 2017. 2017;4:7.

[38] Stout M.J., Zhou Y., Wylie K.M., Tarr P.I., Macones G.A., Tuuli M.G. (2017). Early pregnancy vaginal microbiome trends and preterm birth. Am J Obstet Gynecol.

[39] Sasieni P., Castanon A., Landy R., Kyrgiou M., Kitchener H., Quigley M. (2016). Risk of preterm birth following surgical treatment for cervical disease: executive summary of a recent symposium. BJOG.

[40] Pruski P., MacIntyre D.A., Lewis H.V., Inglese P., Correia G.D.S., Hansel T.T. (2017). Medical swab analysis using desorption electrospray ionization mass spectrometry: a noninvasive approach for mucosal diagnostics. Anal Chem.

