# Supplementary material for: Establishment of vaginal microbiota composition in early pregnancy and its association with subsequent preterm prelabor rupture of the fetal membranes
Source: Transl Res. 2019 May;207:30–43. doi: 10.1016/j.trsl.2018.12.005 (PMC6489901; doi:10.1016/j.trsl.2018.12.005)
Supplement: Supplementary file 1 [file mmc1.docx]

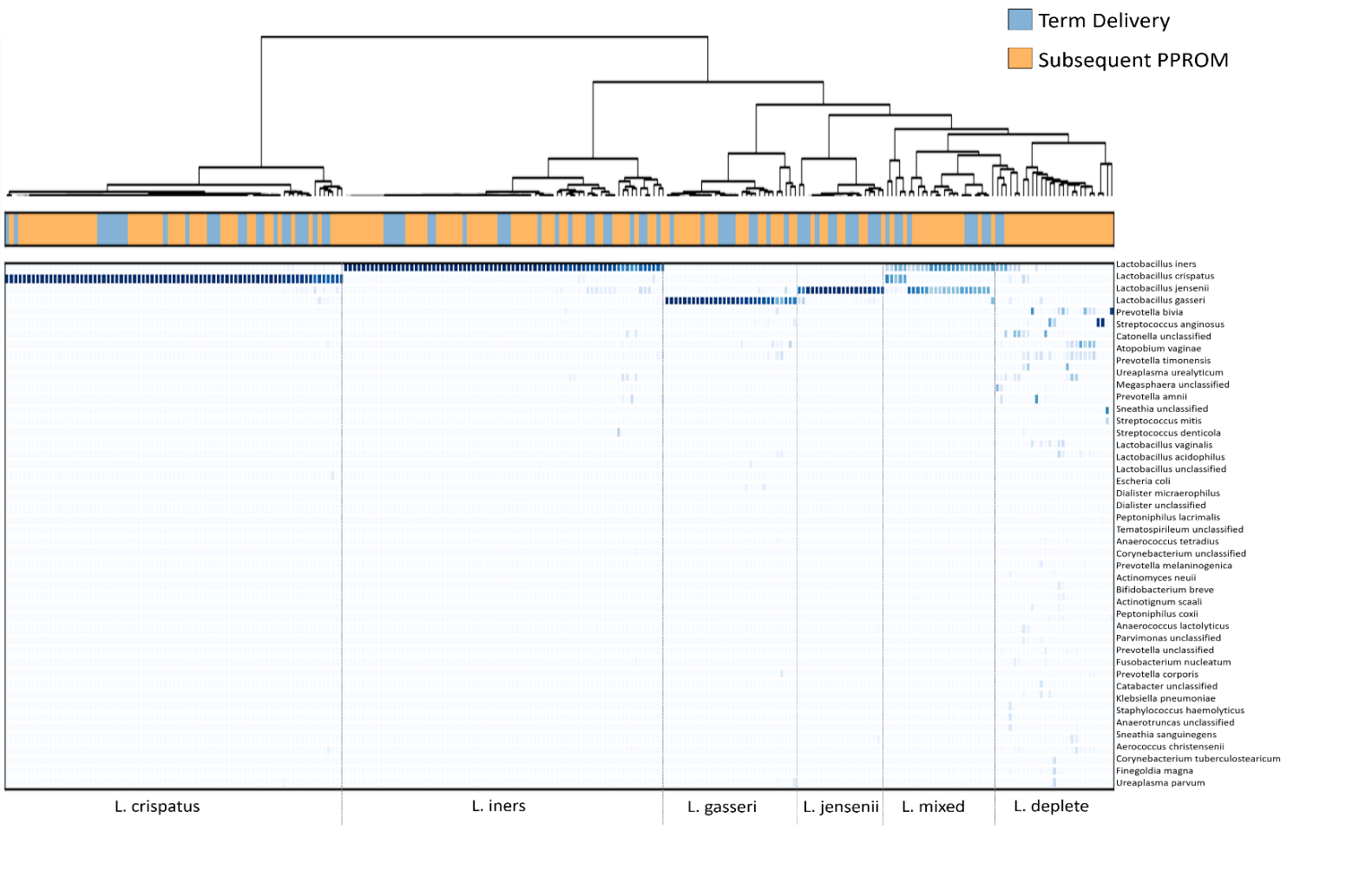


**Supplementary Figure 1: Centroid linkage hierarchical clustering analysis of vaginal bacterial species data from cervical vaginal fluid samples (*n*=254) collected from women with uncomplicated term deliveries (*n*=81, blue) and subsequent PPROM (*n*=173, orange).** Vaginal bacterial communities clustered into *Lactobacillus* dominant (*L.crispatus, L. iners, L. jensenii, L. gasseri, Lactobacillus spp.* mixed and *Lactobacillu*s depleted communities.

**
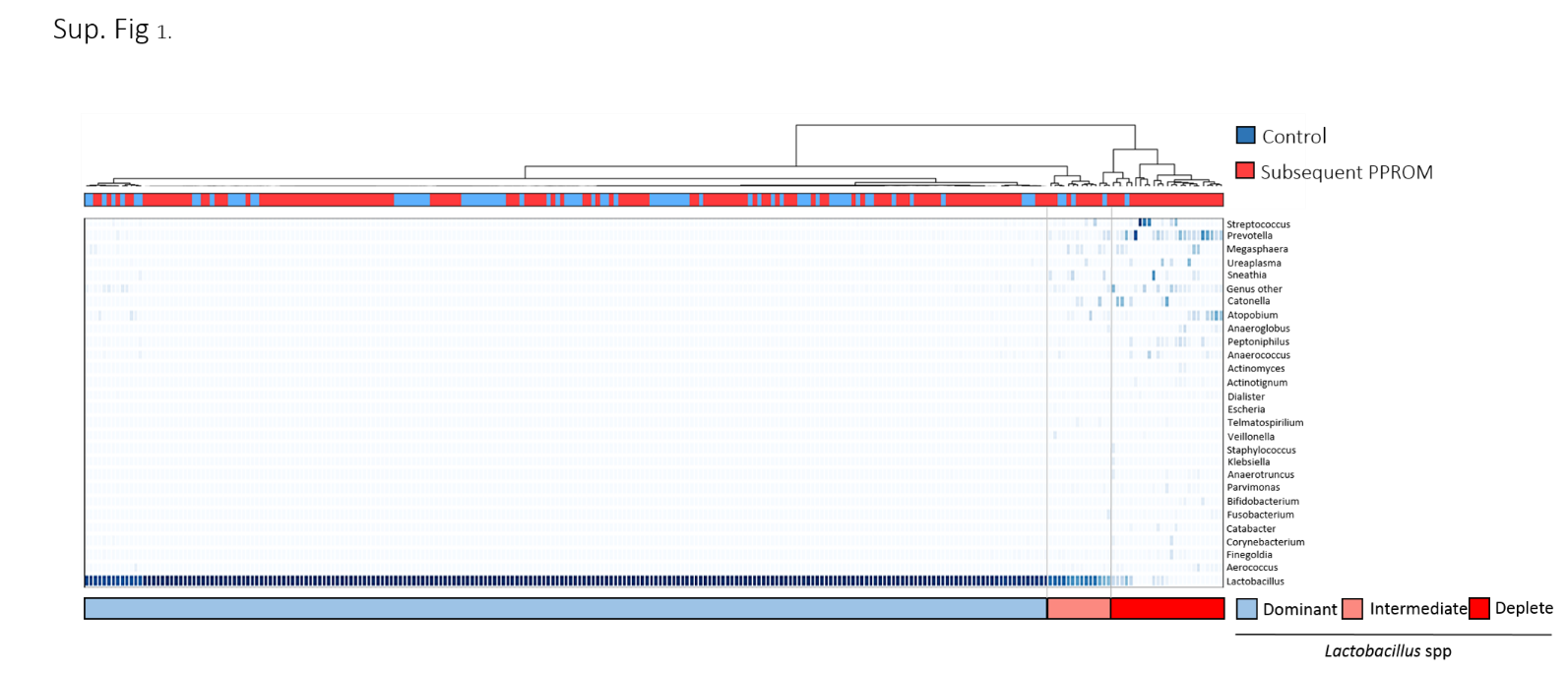
**

**Supplementary Figure 2: Centroid linkage hierarchical clustering analysis of vaginal bacterial genera data from cervical vaginal fluid samples (*n*=254) collected from women with uncomplicated term deliveries (*n*=81, blue) and subsequent PPROM (*n*=173, red).** Vaginal bacterial communities clustered into *Lactobacillus spp.* dominant (75-100%, abundance), intermediate (50-75%, abundance) and deplete (<50% abundance) groups.


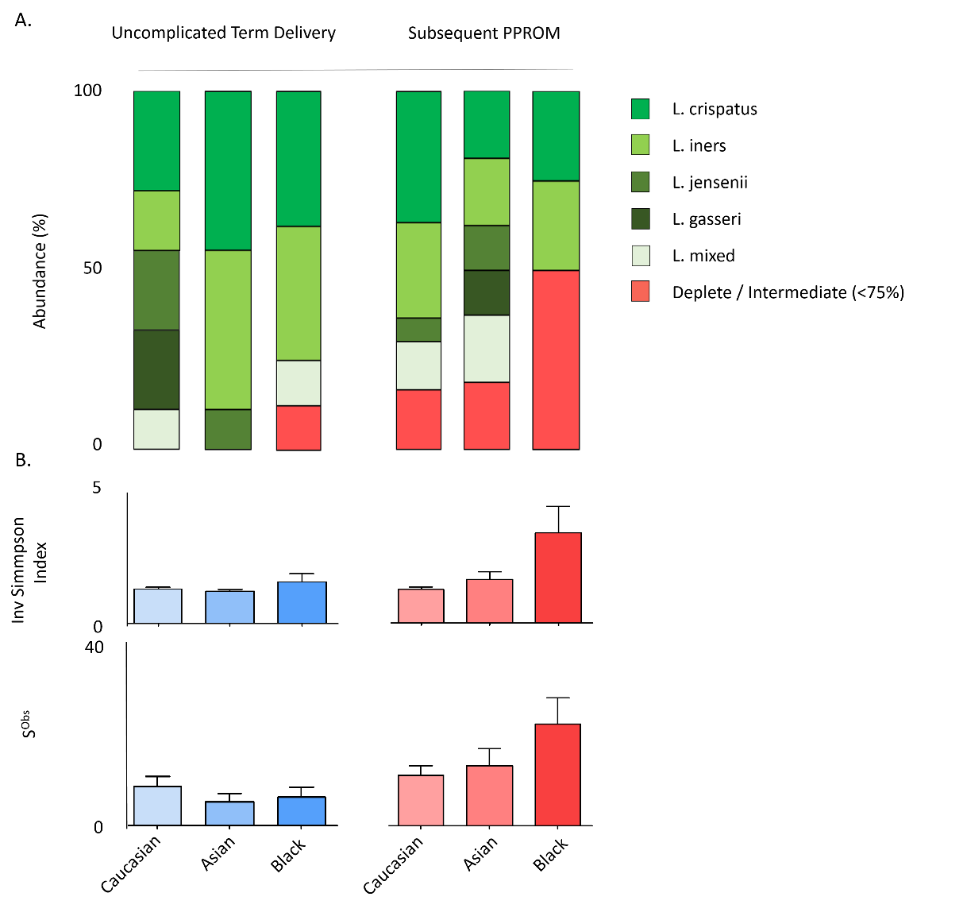


**Supplementary Figure 3: Vaginal bacterial communities and ethnicity for women experiencing subsequent term delivery and PPROM.** **(A)** Proportion of women of Caucasian, Asian and Black ethnicities with a vaginal microbiome dominated by each of the *Lactobacillus spp.* and *Lactobacillus spp.* deplete communities before term delivery and PPROM, sampled at an average gestation of 24^+6^ and 25^+5^ weeks respectively. Black women did not possess *L. gasseri* dominated communities and were the only ethnicity where *Lactobacillus spp.* depletion was observed prior to term delivery. **(B)** Diversity and richness of vaginal bacterial communities for women of Caucasian, Asian and Black ethnicities. Diversity and richness were comparable between women of all ethnicities delivering at term, whilst there was a non-significant trend (P=0.10, Kruskal Wallis, Dunn’s multiple comparison) towards higher richness and diversity amongst black women with subsequent PPROM compared to Caucasian and Asian women.


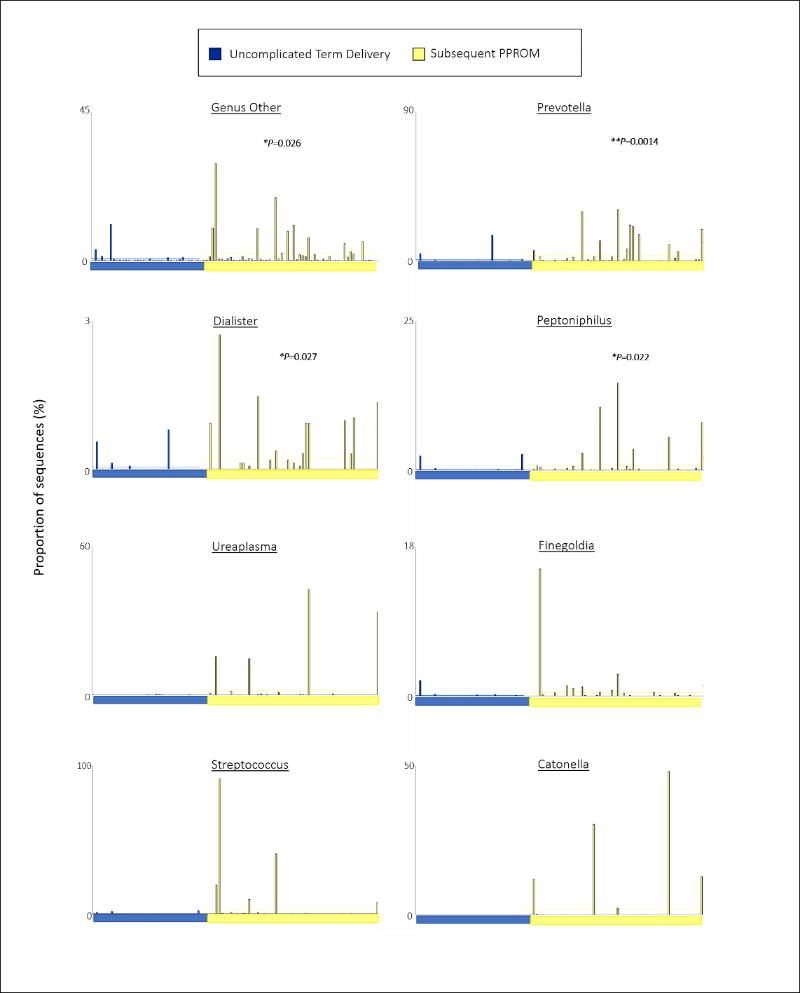


**Supplementary Figure 4:** **Proportion of sequences attributed to bacterial genera differentially expressed between women with subsequent term delivery and PPROM**. *Prevotella, Dialister* and *Peptoniphilus* are significantly higher pre-PPROM (*P=*0.0014, *P=*0.027, *P=*0.022) whilst *Ureaplasma, Finegoldia, Streptococcus* and *Catonella* were more frequently observed pre-PPROM but did not reach statistical significance.


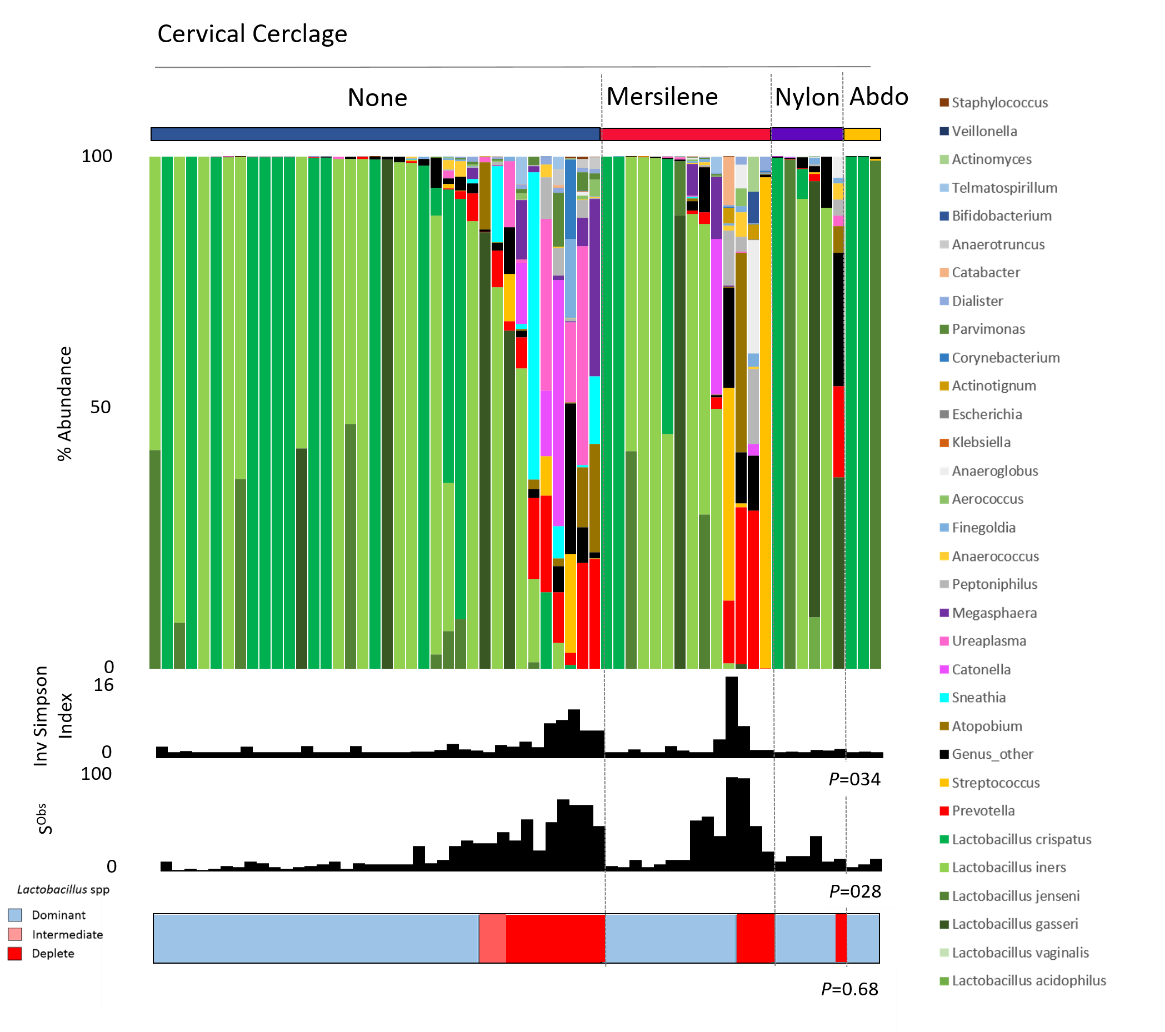


**Supplementary Figure 5: Comparable proportions of women with and without cervical cerclage have a vaginal microbiome deplete in *Lactobacillus spp*.** Stacked Bar Chart displaying percentage abundance of the top 25 bacterial genera, *Lactobacillus spp.*, diversity and richness of vaginal bacterial communities for the last sample taken before PPROM for women with and without cervical cerclage. The number of women with reduced *Lactobacillus spp.* abundance without a cervical cerclage was similar to those with a cervical cerclage (22%, 8/37 vs 26% 6/23, *P*=0.76, Fisher’s exact). Mersilene cerclage was associated with the highest proportion of *Lactobacillus* depletion whilst all of the women with abdominal cerclages had a vaginal microbiome dominated by *Lactobacillus spp.*


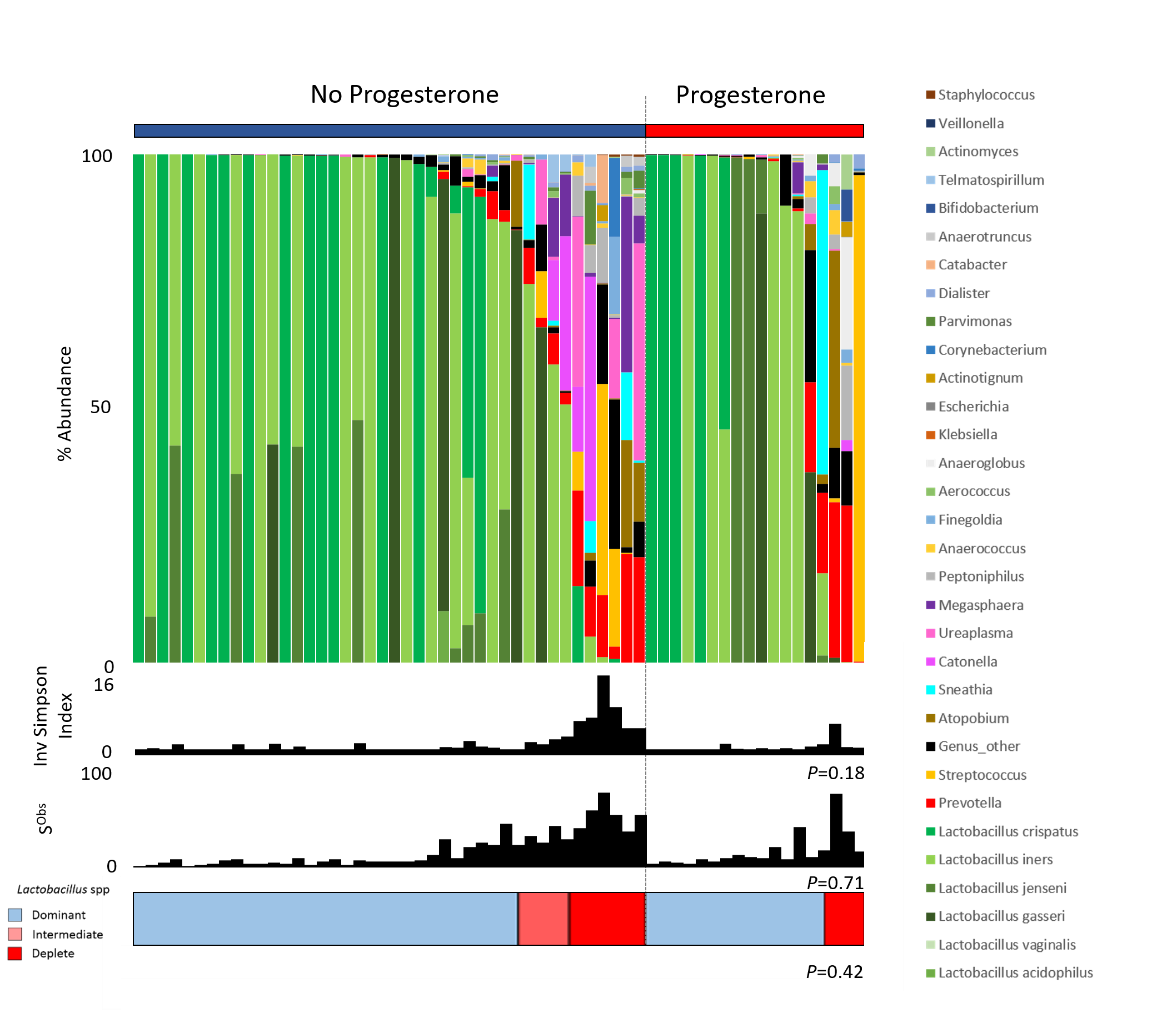


**Supplementary Figure 6:** **Women with vaginal bacterial communities deplete in *Lactobacillus spp*. are equally distributed between those with and without vaginal progesterone treatment.** Percentage abundance of the top 25 bacterial genera, *Lactobacillus spp.*, diversity and richness of vaginal bacterial communities for the last sample taken before PPROM for women with and without progesterone treatment. The number of women with reduced *Lactobacillus spp.* abundance not taking progesterone was similar to those on progesterone treatment (21%, 9/42 vs 28% 5/18, *P*=0.74, Fisher’s exact).

| Pregnancy Outcome | | Term Delivery | | | *P*  value | PPROM | | | *P* value |
| --- | --- | --- | --- | --- | --- | --- | --- | --- | --- |
| Ethnicity | | Black | Asian | Caucasian |  | Black | Asian | Caucasian |  |
| *Lactobacillus* spp abundance | Dominant  (>75%) | 7  (87.5%) | 9  (100%) | 18  (100%) |  | 6  (50%) | 13  (81%) | 25  (83%) |  |
|  | Intermediate  (50-75%) | 0 | 0 | 0 |  | 0 | 0 | 0 |  |
|  | Deplete  (<50%) | 1  (12.5%) | 0 | 0 | 0.22 | 6  (50%) | 3  (19%) | 5  (17%) | 0.08 |
| Dominant bacterial spp. | *L. crispatus* | 3  (37.5%) | 4  (44.5%) | 5  (28%) |  | 3  (25%) | 3  (19%) | 11  (36%) |  |
|  | *L. iners* | 3  (37.5%) | 4  (44.5%) | 3  (17%) |  | 3  (25%) | 3  (19%) | 8  (26%) |  |
|  | *L. gasseri* | 0 | 0 | 4  (22%) |  | 0 | 2 (12%) | 0 |  |
|  | *L. jensenii* | 0 | 1  (11%) | 4  (22%) |  | 0 | 2  (12%) | 2  (7%) |  |
|  | L.Mixed | 1  (12.5%) | 0 | 2  (11%) |  | 0 | 3  (19%) | 4  (14%) |  |
|  | Non Lactobacilli | 1  (12.5%) | 0 | 0 | 0.32 | 6  (50%) | 3  (19%) | 5  (17%) | 0.14 |
| Bacterial  Richness /Diversity | SOBs | 6.6  (2.1) | 5.6  (1.9) | 9.1  (2.3) | 0.84 | 24.2  (6.2) | 14.4 (4.0) | 12.1  (2.2) | 0.10 |
|  | Inverse Simpson  Index | 1.56  (0.32) | 1.22 (0.07) | 1.29 (0.08) | 0.39 | 3.37  (1.01) | 1.64 (0.27) | 1.27 (0.08) | 0.18 |

**Supplementary Table 1.** Distribution of *Lactobacillus* spp. dominant and deplete communities across ethnicity for women with subsequent term deliveries and PPROM

SOB- Species Observed, P values: *Lactobacillu*s spp. abundance and dominant *Lactobacillus* spp- Fisher’s exact, Bacterial Diversity indices-Kruskal Wallis with Dunn’s multiple comparison test.

**Supplementary Table 2.** Bacterial richness and diversity at each gestational time window for subsequent term deliveries and PPROM

| Gestation (wks) | SOBS | | *P* value | Inverse Simpson | | *P* value |
| --- | --- | --- | --- | --- | --- | --- |
|  | TD | PP |  | TD | PP |  |
| 6-11^+6^ | 10.7 (3.6) | **9.2 (2.8)**** | 0.22 | 1.35 (0.13) | 1.65 (0.36) | 0.39 |
| 12-17^+6^ | **15.7 (5.4)*** | 10.3 (1.9) | 0.22 | 1.41 (0.16) | 1.66 (0.16) | 0.72 |
| 18-23^+6^ | 8.1 (1.9) | 12.8 (1.8) | 0.16 | 1.42 (0.19) | 1.84 (0.26) | 0.98 |
| 24-29^+6^ | **7.6 (2.0)*** | **20.1 (4.8)**** | 0.12 | 1.30 (0.07) | 2.00 (0.31) | 0.81 |
| 30-36^+6^ | 9.4 (3.0) | 13.5 (3.4) | 0.95 | 1.29 (0.12) | 2.03 (0.59) | 0.56 |

TD- Term Delivery, PP- Pre-PPROM, wks- weeks. Mean (standard error), P value: Mann Whitney. *Term Delivery SOB significantly higher at 12-17^+6^ compared to 24-29^+6^ weeks (P=0.03, Mann Whitney), ** Subsequent PROM SOB significantly higher at 24-29^+6^ compared to 6-11^+6^ weeks (P=0.02, Mann Whitney
